# Expert consensus on the reporting and clinical follow up of individuals with incidentally discovered germline *RET* variants in the UK

**DOI:** 10.1530/ERC-26-0194

**Published:** 2026-09-02

**Authors:** Helen Hanson, Katie Snape, Renuka P Dias, Soo-Mi Park, M Guftar Shaikh, Tom R Kurzawinski, Louise Izatt, Kashyap A Patel, Márta Korbonits, Ruth T Casey

**Affiliations:** ^1^ South West Clinical Genetics Service, Royal Devon University Healthcare NHS Foundation Trust, Exeter, UK; ^2^ Department of Clinical and Biomedical Sciences, University of Exeter Medical School, Exeter, UK; ^3^ Southwest Thames Regional Genetics Service, St George’s Hospital, London, UK; ^4^ Immunology and Immunotherapy, College of Medicine and Health, University of Birmingham, Birmingham, UK; ^5^ Department of Paediatric Endocrinology and Diabetes, Birmingham Women’s and Children’s Hospital NHS Foundation Trust, Birmingham, UK; ^6^ Department of Genomic Medicine, Cambridge University Hospitals NHS Foundation Trust, Cambridge, UK; ^7^ Department of Endocrinology, Royal Hospital for Children, Glasgow, UK; ^8^ Centre for Endocrine Surgery, Great Ormond Street and University College Hospitals, NHS Foundation Trusts, London, UK; ^9^ Department of Clinical Genetics, Guy’s and St Thomas’ NHS Foundation Trust, London, UK; ^10^ Centre for Endocrinology, William Harvey Research Institute, Barts and The London School of Medicine and Dentistry, Queen Mary University of London, London, UK; ^11^ Department of Endocrinology, Cambridge University Hospitals NHS Foundation Trust, Cambridge, UK; ^12^ Department of Genetics, University of Cambridge, Cambridge, UK

**Keywords:** oncogene, medullary thyroid cancer, genetic counselling

## Abstract

Incidentally discovered pathogenic germline genetic variants refer to the finding of a pathogenic variant in a gene that is unrelated to the reason for the initial test and is not actively sought. Our clinical understanding of the risk of developing a particular medical condition and the required clinical action for a specific pathogenic gene variant is predominantly based on knowledge and information acquired from cases ascertained through a ‘phenotype-first approach’ rather than in clinically unselected individuals. Therefore, a modified approach is required for incidentally discovered gene variants. Data from large UK and US population-based cohorts have demonstrated that *RET* variants classified as moderate-risk *RET* variants as per the American Thyroid Association (ATA) classification have a low penetrance for medullary thyroid cancer and other *RET*-related conditions (e.g. phaeochromocytoma) and are not associated with excess mortality when identified incidentally in clinically unselected adult individuals. Here, we provide guidance based on multidisciplinary expert consensus opinion for the reporting and subsequent clinical surveillance and management of patients with incidentally discovered *RET* gene variants in the UK.

## Introduction

The penetrance for cancers in individuals incidentally identified to harbour pathogenic variants in cancer susceptibility genes (CSGs) is not well understood. However, multiple studies have demonstrated lower penetrance of monogenic pathogenic variants, including CSGs in population-based cohorts, than in cases clinically ascertained due to the manifestation of the disease (1, 2, 3, 4). There are likely to be several factors that influence penetrance including the distribution of modifying genes and potential environmental influences (5). In the UK, all patients offered broad genetic testing by whole-exome sequencing (WES) or whole-genome sequencing (WGS) should be carefully counselled on the risk of incidental findings prior to genetic testing and reporting, and the discussion should be documented as part of standard ‘record of discussion’ forms available through the local genomic laboratory hub websites. In this article, we will consider this problem specifically for the *RET* gene.

### Genotype–phenotype correlations for *RET*

Long-term observational studies have facilitated an understanding that specific *RET* variants cause more severe phenotypes, for example, the age of onset and severity of MTC or the age of onset of phaeochromocytoma. This has facilitated the classification of specific variants in the *RET* gene according to the associated severity and penetrance of the most penetrant phenotype: MTC. The American Thyroid Association has classified *RET* variants into three categories: highest, high and moderate (Table 1) (6, 7). The 10-year survival of patients with high-risk *RET* variants is 75.5% compared to patients with moderate-risk variants, where 10-year survival is 97.4% (8).

**Table 1:** Classification of *RET* variants.

ATA risk category	Variants (amino acid change)	Phenotype	Timing of risk-reducing thyroidectomy as per ATA guidelines (6)
Highest*	M918T	**MEN2B** MTC Phaeochromocytoma Other: marfanoid body habitus, pes cavus, pectus excavatum, high-arched palate, scoliosis) and gastrointestinal tract ganglioneuromatosis	6 months–1 year
High*	C634F/G/R/S/W/Y A883F Double variants: V804M/Y806C V804M/E805K V804M/S904C V804M/Q781R Y791F/M918T M918T/S922Y	**MEN2A** MTC Phaeochromocytoma HPTH Other: cutaneous lichen amyloidosis, Hirschprung's disease	∼5 years
Moderate	G533C C609F/G/R/S/Y C611F/G/S/Y/W C618F/R/S C620F/R/S C630R/Y D631Y K666E/N E768D L790F V804L/M S891A	**MEN2A** MTC Phaeochromocytoma HPTH Other: cutaneous lichen amyloidosis, Hirschprung's disease	Timing is determined by rising calcitonin levels

* These variants, if detected incidentally, should be reported.

ATA, American Thyroid Association; MTC, medullary thyroid cancer; HPTH, primary hyperparathyroidism.

Bold text indicates the genetic syndrome.

The recommendations for surveillance rather than an age-defined timing of risk-reducing surgery for individuals with moderate-risk *RET* variants were based on the observation of variable penetrance of moderate-risk *RET* variants, even within the same family. The moderate-risk *RET* variant (c.2410G>A p.(Val804Met)) is the most frequently observed pathogenic *RET* variant, and this variant has also been noted at higher-than-expected frequency in large-scale genomic databases (9). Notably, the penetrance of MTC for carrier relatives of Val804Met-positive cases was recently calculated as 4% by age 70 years (10), suggesting variable penetrance of certain *RET* variants even within families affected by MEN2A and aligns with reports of a milder phenotype associated with *RET* Val804Met and Val804Leu variants within MEN2A families, including a later penetrance and indolent MTC (11).

### Penetrance of *RET* in clinically unselected populations

The ATA classification and corresponding guidance was published in 2015 (6) when genetic testing of *RET* would typically have been prompted by a known family history of MEN2 and/or MTC, rather than identification through broader genetic testing for unrelated reasons. The estimated prevalence of MEN2 is 1 in 30,000 (0.003%) to 50,000 (0.005%) (12). However, two recent studies have identified pathogenic *RET* variants in three unselected population cohorts (UK Biobank) and two United States health system-based population cohorts (Geisinger MyCode and ‘All of Us’) with a prevalence of 0.04, 0.06 and 0.04%, respectively (13, 14). The first study by West *et al.* investigated the penetrance of MTC and mortality rates in incidental *RET* variant carriers in two unselected patient cohorts (UK Biobank and Geisinger MyCode) and compared these results to a clinically selected cohort (patients with a personal or family history of MEN2) from a single institution in the UK (Exeter) (13).

Most identified *RET* variants in these studies were moderate-risk *RET* variants. The risk of MTC by age 75 years in the UK Biobank cohort was calculated as 2.2, and 19.3% in the US Geisinger MyCode cohort (13). Most individuals in the UK Biobank cohort had not undergone thyroidectomy (98.2%), and the all-cause mortality rate in this cohort was comparable to that of non-*RET* carriers (13). Furthermore, the penetrance of the next most penetrant MEN2-associated condition, phaeochromocytoma, was also noted to be very low in both population datasets (1/169 (0.6%) patients in the UK Biobank cohort and 2/77 (2.6%) in the US Geisinger MyCode population dataset) (13). All patients identified with phaeochromocytoma had a high-risk *RET* variant (13). Importantly, this study by West *et al.* did not include young adults or paediatric cases and included very few cases of high- or highest risk *RET* variants (13). The mean age at recruitment in all three clinical cohorts (UK Biobank, Geisinger MyCode cohort and the Exeter clinical cohort) in this study was >45 years (13). However, the age-related penetrance for moderate-risk *RET* variants in the clinically selected Exeter cohort was 16% by age 30 years in this study versus 0% by age 30 years in the unselected UK Biobank cohort (12). Data from these two studies, including three clinically unselected population-based datasets (13, 14), suggest that the prevalence of moderate-risk *RET* gene variants is much higher than the prevalence of MEN2 in the general population. Furthermore, the low penetrance of MTC in these cohorts suggests that a tailored approach to surveillance and risk-reducing thyroidectomy is required for patients with incidentally discovered *RET* gene variants due to a significant risk of overtreatment. Notably, based on this robust recent data, only the highest risk *RET* gene variant p.(Met918Thr) will be reported as part of the newborn genomic screening in the UK Generation Study from March 2026 (https://www.genomicsengland.co.uk/initiatives/newborns). This specific *RET* variant was included as it occurs as a *de novo* event in 90% of cases and is associated with the highest reported penetrance of MTC and clinical actionability.

### Current UK guidance on incidentally discovered pathogenic gene variants

The British Society for Genomic Medicine (BSGM) has provided recommendations for UK genomic laboratories on the reporting of clinically relevant incidental findings (15). This guidance recommends that pathogenic (class V) variants in genes unrelated to the referral reason can be reported in the absence of a clinical phenotype, if there is evidence of high penetrance and available treatment or surveillance that is likely to improve clinical outcomes for the individual or family (15). The American College of Medical Genetics and Genomics has issued an agreed list of 28 ‘clinically actionable’ CSGs, which it recommends being reported should an incidentally discovered pathogenic variant in one of these genes be identified (16). Included in this list are nine CSGs, which specifically predispose to endocrine tumours or endocrine neoplasia syndromes: *MAX, MEN1, RET, SDHB, SDHC, SDHD, SDHAF2, TMEM127 and VHL.* In 2023, a cancer genetics expert group within the European Society for Medical Oncology also provided a list of seven ‘most actionable’ genes in the context of incidentally discovered pathogenic (class V) or likely pathogenic (class IV) variants on tumour-only sequencing, including *RET*. This guidance recommends that a (likely) pathogenic variant in one of these seven genes on tumour-only sequencing should prompt follow-up germline genetic testing in all tumour types (17). However, a later 2025 UK Cancer Genetics Group (UKCGG) position statement (18) advised that only highest and high-risk *RET* variants detected on sequencing in non-*RET*-associated tumours should prompt follow-up germline genetic testing, due to the emerging data on the reduced penetrance of moderate-risk variants ascertained outside the context of a relevant family history (13, 14). An updated version of the BSGM guidance on incidental findings, published in January 2026, has also provided further advice regarding specific incidentally discovered cancer susceptibility genes, including *RET*, and recommended that only high- and highest risk *RET* variants (gain-of-function variants known to lead to MEN2) should be reported (15).

In this consensus document, we outline suggestions for the reporting of incidentally identified germline *RET* variants, clinical surveillance strategies and the role and timing of risk-reducing thyroidectomy for individuals with incidentally discovered *RET* variants. These suggestions are based on expert consensus opinion, anecdotal case reports and limited high-quality data.

## Methods

A UK-based working group of 10 experts across the disciplines of medical genetics, endocrine surgery and adult and paediatric endocrinology was assembled, and agreement was reached on 16 statements of recommendation. As is typical for most rare diseases, the volume of peer-reviewed high-quality evidence available to consider for these guidelines was limited. To balance the weight of both published evidence with the wealth of expert experience and knowledge, we have used a scale for evidence grading that was introduced in the European Reference Network (ERN) GENTURIS Cancer Surveillance Guideline (19) for individuals with PTEN hamartoma tumour syndrome: i) strong evidence: consistent evidence and new evidence unlikely to change recommendation and expert consensus; ii) moderate evidence: expert consensus or majority decision but with inconsistent evidence or significant new evidence expected; and iii) weak evidence: inconsistent evidence and limited expert agreement. Importantly, when interpreting the grading of evidence, the authors want to emphasise that a lack of high-quality evidence does not mean that the evidence available is poor, it just means that the evidence is limited, which further emphasises the need for expert consensus opinion to guide management.

The recommendations were then endorsed by the following learned societies: UK Cancer Genetics Group (UKCGG), UK Clinical Genetics Society (UKCGS), Society for Endocrinology (SFE), British Society for Paediatric Endocrinology and Diabetes (BSPED), British Association of Endocrine and Thyroid Surgeons (BAETS) and patient advocacy group AMEND (https://www.amend.org.uk).

### Recommendations

***R1. We recommend that only the highest and* *high-* *risk* *RET variants (* Table 1 *)* *are reported when identified incidentally during germline genetic testing in the following settings:***

Grade of evidence: (2) moderate evidence: expert consensus or majority decision but with inconsistent evidence or significant new evidence expected.

- *The germline genetic test was undertaken for a disorder causally unrelated to any MEN2 phenotype (*e.g. *intellectual disability, epilepsy, neurogenerative disorders or ataxia).*
- *There is no known personal or family history of any MEN2-related conditions.*

***R2. We do not recommend reporting moderate-risk RET variants or delaying reporting to ask clinicians to clarify personal or family history. We understand that in most circumstances, a detailed and specific family history may not be available to scientists or clinicians. If an MEN2-related personal or family history is noted at the time of reporting in the referral details, specialist genetics advice should be sought with respect to reporting moderate-risk RET variants under these circumstances.***

Grade of evidence: (2) moderate evidence: expert consensus or majority decision but with inconsistent evidence or significant new evidence expected.

***R3. We recommend referral to specialised regional genetics services for all patients identified with an incidental, highest and high-risk RET variant or a moderate-risk RET variant if an MEN2-related personal or family history has been noted, to ensure detailed and complete assessment of the personal and family history, contextualised discussions around risk, implications for the wider family and reproductive decision-making and entry to the National Inherited Cancer Predisposition Register.***

Grade of evidence: (2) moderate evidence: expert consensus or majority decision but with inconsistent evidence or significant new evidence expected.

***R4. We recommend offering cascade predictive genetic testing to relatives for the highest and high-risk RET variants reported through these pathways through specialised regional genetics services. This will ensure the following:***

- ***Detailed and complete assessment of the personal and family history and contextualised discussions around risk.***
- ***Timely referrals to an endocrinologist for appropriate screening and management in those individuals who test positive for the familial RET variant.***
- ***Appropriate discussions around available reproductive choices including non-invasive/invasive prenatal genetic testing and pre-implantation genetic testing for monogenic disorders (PGT-M).***

Grade of evidence: (2) moderate evidence: expert consensus or majority decision but with inconsistent evidence or significant new evidence expected.

Where moderate-risk *RET* variants have been reported due to an identified related family history, it is essential to seek specialist genetics advice to ensure appropriate management of the wider family.

Where incidental moderate-risk *RET* variants have historically been reported and new family members present for consideration of cascade predictive genetic testing, we would recommend a review of the family history for any MEN2-related features. Where there is no relevant family history, we would advise that cascade testing is not routinely offered and that patients are counselled on the low penetrance for incidentally discovered moderate-risk *RET* variants (15) (see R15).

**Figure 1:**
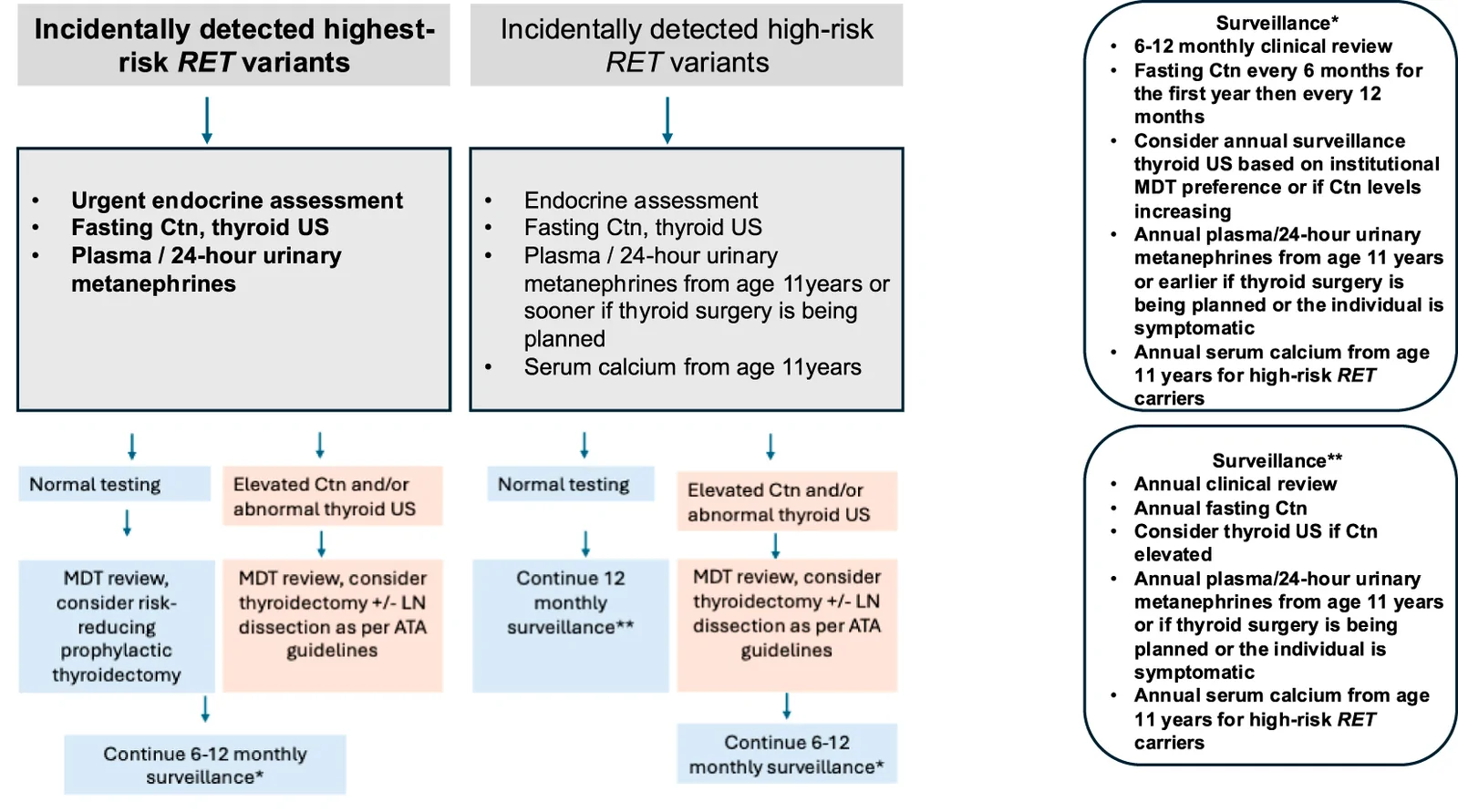
Clinical screening guidance for patients with incidentally discovered highest and high-risk *RET* variants. ATA, American Thyroid Association; Ctn, calcitonin; LN, lymph node; Thyroid US, thyroid ultrasound.

***R5. We advise that all paediatric and adult patients with an incidentally discovered highest risk RET variant are offered urgent endocrine assessment including fasting serum calcitonin and a thyroid ultrasound. Risk-reducing thyroidectomy should be considered in children and adults with an incidentally discovered highest risk RET variant after multidisciplinary team (MDT) review even if baseline testing is normal. Plasma metanephrines or 24-h urinary metanephrines should be tested from the age of 11 years or earlier if there are symptoms of catecholamine excess or thyroid surgery is planned (Fig. 1* *).***

Grade of evidence: (1) strong evidence: consistent evidence and new evidence unlikely to change recommendation and expert consensus.

In patients with an incidentally found highest risk *RET* variant associated with MEN2B, expert consensus is that thyroidectomy is prioritised given the 100% penetrance of MTC and the high rate of *de novo* mutations with this variant.

It is advised that a thyroid ultrasound and calcitonin are measured at first assessment for patients with an incidentally discovered highest risk *RET* variant to provide presurgical anatomical information and a baseline calcitonin measurement.

***R6. We advise that all adult and paediatric patients with incidentally discovered high-risk RET variants are offered rapid endocrine assessment including fasting serum calcitonin and a thyroid ultrasound. Plasma metanephrines or 24-h urinary metanephrines should be tested from the age of 11 years or earlier if there are symptoms of catecholamine excess or thyroid surgery is planned. Serum calcium should be checked from age 11 years. Serum calcitonin should be checked annually from the age of 5 years. Thyroidectomy should be considered in patients with abnormal or rising fasting calcitonin or sonography findings (grade of evidence: moderate).***

Grade of evidence: (1) strong evidence: consistent evidence and new evidence unlikely to change recommendation and expert consensus.

For patients with incidentally discovered high-risk *RET* variants, a thyroid ultrasound and calcitonin are advised at first assessment to assess whether surgery is indicated, or ongoing surveillance can be considered. We advise that thyroidectomy is considered if there is abnormal or rising fasting calcitonin and/or suspicious findings for MTC on a thyroid ultrasound. We have not advised immediate risk-reducing surgery as there is limited information about the penetrance of incidentally discovered high-risk *RET* variants and therefore the value versus risk of risk-reducing surgery in patients with incidentally discovered high-risk *RET* variants. All patients with incidentally discovered high-risk *RET* variants should be discussed at a specialist MDT before proceeding with surgery. We acknowledge that this advice may change as more information becomes available. In the interim, we recommend careful counselling of patients and expert MDT review of individual patients and pedigrees with high-risk *RET* variants.

***R7. We advise that all adult and paediatric patients with incidentally discovered highest and high-risk RET variants who have undergone risk-reducing thyroid surgery are offered 6-monthly endocrine assessment including 6-monthly fasting serum calcitonin for the first year (subsequently annual calcitonin if it remains undetectable post-operatively) and 12-monthly plasma metanephrines or 24-h urinary metanephrines and serum calcium (for high-risk only) from the age of 11 years. Plasma metanephrines or 24-h urinary metanephrines should be screened for earlier if there are symptoms of catecholamine excess. Interval thyroid ultrasound can be considered based on institutional preference or if serum calcitonin becomes elevated.***

Grade of evidence: (1) strong evidence: consistent evidence and new evidence unlikely to change recommendation and expert consensus.

***R8. We advise that all adult and paediatric patients with incidentally discovered high- or highest risk RET variants found to have an elevated calcitonin or abnormal thyroid ultrasound are reviewed at a thyroid cancer MDT. Consideration should be given to a total thyroidectomy ± lymph node dissection, if lymph nodes are noted to be enlarged clinically or on ultrasound or if metastatic nodal disease is detected cytologically preoperatively.***

Grade of evidence: (1) strong evidence: consistent evidence and new evidence unlikely to change recommendation and expert consensus.

***R9. We advise that all adult and paediatric patients with incidentally discovered highest or high-risk RET variants who have a serum calcitonin concentration >500 ng/L are considered for full-body staging using CT/MRI or molecular imaging based on institutional preference ahead of a planned thyroidectomy to screen for metastatic disease.***

Grade of evidence: (1) strong evidence: consistent evidence and new evidence unlikely to change recommendation and expert consensus.

**R10. We advise that all adult and paediatric patients with an incidentally discovered highest and high-risk RET gene variant who have undergone a thyroidectomy for confirmed MTC undergo long-term surveillance for recurrence of MTC in line with American Thyroid Association guidance (6)*.***

Grade of evidence: (1) strong evidence: consistent evidence and new evidence unlikely to change recommendation and expert consensus.

***R11. We advise that all patients with incidentally discovered high-risk RET variants who have a normal first endocrine assessment are offered 12-monthly endocrine assessment including fasting serum calcitonin and plasma metanephrines or 24-h urinary metanephrines and serum calcium from the age of 11 year. Plasma metanephrines or 24-h urinary metanephrines should be screened for earlier if there are symptoms of catecholamine excess or thyroid surgery is planned. Interval thyroid ultrasound can be considered based on institutional preference or if serum calcitonin becomes elevated.***

Grade of evidence: (2) moderate evidence: expert consensus or majority decision but with inconsistent evidence or significant new evidence expected.

***R12. We advise against the reporting of incidentally discovered moderate-risk RET variants, but we acknowledge that there will be a cohort of patients for whom an incidentally discovered moderate-risk RET variant has historically been reported and for whom clinical surveillance may have been advised. We would recommend that clinical review including an up-to-date assessment of personal and family history can be considered for these patients to facilitate further discussion and counselling.***

Grade of evidence: (2) moderate evidence: expert consensus or majority decision but with inconsistent evidence or significant new evidence expected.

In the UK, the National Genomic Test Directory outlines specific phenotype-driven genetic testing, making it less likely that patients with an MEN2 phenotype could be incorrectly classified as harbouring an incidental *RET* variant. Current indications for germline genetic testing for MEN2 include: MTC at any age, ≥2 MEN2-related endocrine abnormalities (at any age) or ≥1 MEN2-related endocrine abnormality and a first-degree relative with ≥1 MEN2-related endocrine abnormality. *RET* is also included on gene panels related to primary hyperparathyroidism (R151 indication) and phaeochromocytoma (R223 indication) (https://www.england.nhs.uk/publication/national-genomic-test-directories).

***R13. We advise that all adult and paediatric patients who have historically received the diagnosis of an incidentally discovered moderate-risk RET variant are counselled on the low penetrance for incidentally discovered moderate-risk RET variants. If the first endocrine assessment is normal, we would advise discussion at an expert MDT and with the patient regarding the need for further long-term endocrine surveillance and follow-up. We advise against continued longitudinal surveillance in the absence of a personal or family history of MEN2 or abnormal results at first assessment for patients who have historically received a diagnosis of an incidentally discovered moderate-risk RET variant.***

Grade of evidence: (2) moderate evidence: expert consensus or majority decision but with inconsistent evidence or significant new evidence expected.

**R14. For adult and paediatric patients, who have historically received the diagnosis of an incidental moderate-risk RET variant prior to this recommendation and for whom a family or personal history of MEN2 becomes apparent during clinical review, or for adult and paediatric patients who are identified with an incidental moderate-risk RET variant, which is reported because of a known personal or family history of MEN2 at the time of genetic testing, we advise longitudinal follow-up in line with ATA recommendations for moderate-risk RET variants (6)*.***

Grade of evidence: (2) moderate evidence: expert consensus or majority decision but with inconsistent evidence or significant new evidence expected.

***R15. We advise that all adult and paediatric patients with an incidentally discovered highest and high-risk RET gene variant found to have elevations in plasma metanephrines or 24-h urinary metanephrines are reviewed at an adrenal MDT and consideration is given to dedicated adrenal imaging* *(* *CT or MRI* *)* *.***

Grade of evidence: (1) strong evidence: consistent evidence and new evidence unlikely to change recommendation and expert consensus.

**R16. We advise that all adult and paediatric patients with any incidentally discovered pathogenic RET gene variant diagnosed with a phaeochromocytoma are managed as per existing guidance for RET-associated phaeochromocytoma (6, 20).**

Grade of evidence: (1) strong evidence: consistent evidence and new evidence unlikely to change recommendation and expert consensus.

### Summary

Based on existing data for incidentally discovered germline *RET* variants in the context of what is also known about the reduced penetrance of other incidentally discovered CSGs, it would seem prudent that tailored strategies for reporting, subsequent surveillance and risk-reducing interventions for individuals with incidentally discovered *RET* variants are adopted. The authors acknowledge that there are limited real-world clinical data currently available to further support these recommendations, but this guidance aligns with recently published UK guidance for the reporting of incidentally discovered CSG variants and the recognition of the need for tailored strategies where CSG variants are identified outside phenotype-driven testing. This guidance and the updated BSGM guidance have taken into consideration the recent independent population-based studies, which have both demonstrated a very low penetrance of MTC in clinically unselected cohorts with moderate-risk *RET* variants (13, 14).

All patients with incidentally discovered highest and high-*RET* gene variants require genetic counselling, MDT review and referral to experienced endocrinology and endocrine surgery centres. We have also provided guidance on the follow-up of individuals and families for whom an incidentally discovered moderate-risk *RET* variant has been historically reported and advise that further follow-up and repeat counselling is offered to these families. Moving forward, priority should be given to gathering cases at a national and international level to better guide future management of patients with incidentally discovered variants in cancer susceptibility genes. The addition of patients with MEN2-related *RET* variants to the UK National Inherited Cancer Predisposition Register (https://digital.nhs.uk/ndrs/our-work/genomics/nicpr) should facilitate longitudinal studies to refine evidence-based recommendations over time.

## Declaration of interest

RPD has received honoraria from Sanofi (participation in advisory board for Teplizumab) and speaker fees from Sanofi and Sandoz. Márta Korbonits is Deputy Editor of Endocrine-Related Cancer. Márta Korbonits was not involved in the review or editorial process for this paper, on which she is listed as an author.

## Funding

This research did not receive any specific grant from any funding agency in the public, commercial or not-for-profit sector.
